# Development and testing of an implementation strategy for a complex housing intervention: protocol for a mixed methods study

**DOI:** 10.1186/s13012-014-0138-4

**Published:** 2014-10-17

**Authors:** Dennis P Watson, Jeani Young, Emily Ahonen, Huiping Xu, Macey Henderson, Valery Shuman, Randi Tolliver

**Affiliations:** Department of Health Policy and Management, Indiana University, Richard M. Fairbanks School of Public Health, Indiana University-Purdue University Indianapolis, 714 N. Senate Ave, Indianapolis, IN 46202 USA; University Information and Technology Services, Indiana University-Purdue University Indianapolis, 535 W. Michigan St, Indianapolis, IN 46202 USA; Department of Social Behavioral Sciences & Department of Environmental Health Science, Indiana University, Richard M. Fairbanks School of Public Health, Indiana University-Purdue University Indianapolis, 714 N. Senate Ave, Indianapolis, IN 46202 USA; Department of Biostatistics, Indiana University, Richard M. Fairbanks School of Public Health, Indiana University-Purdue University Indianapolis, 714 N. Senate Ave, Indianapolis, IN 46202 USA; Midwest Harm Reduction Institute, Heartland Health Outreach Inc., 1207 W. Leland Ave., Chicago, IL 60640 USA

**Keywords:** Implementation strategy, Protocol, Housing, Housing First, Fidelity, Training, Technical assistance

## Abstract

**Background:**

There is currently a lack of scientifically designed and tested implementation strategies. Such strategies are particularly important for highly complex interventions that require coordination between multiple parts to be successful. This paper presents a protocol for the development and testing of an implementation strategy for a complex intervention known as the Housing First model (HFM). Housing First is an evidence-based practice for chronically homeless individuals demonstrated to significantly improve a number of outcomes.

**Methods/design:**

Drawing on practices demonstrated to be useful in implementation and e-learning theory, our team is currently adapting a face-to-face implementation strategy so that it can be delivered over a distance. Research activities will be divided between Chicago and Central Indiana, two areas with significantly different barriers to HFM implementation. Ten housing providers (five from Chicago and five from Indiana) will be recruited to conduct an alpha test of each of four e-learning modules as they are developed. Providers will be requested to keep a detailed log of their experience completing the modules and participate in one of two focus groups. After refining the modules based on alpha test results, we will test the strategy among a sample of four housing organizations (two from Chicago and two from Indiana). We will collect and analyze both qualitative and quantitative data from administration and staff. Measures of interest include causal factors affecting implementation, training outcomes, and implementation outcomes.

**Discussion:**

This project is an important first step in the development of an evidence-based implementation strategy to increase scalability and impact of the HFM. The project also has strong potential to increase limited scientific knowledge regarding implementation strategies in general.

## Background

There is currently a lack of theoretically based, well-described, and testable implementation strategies [1,2]. Strongly designed implementation strategies are particularly important for the implementation of complex interventions—meaning interventions “consisting of multiple behavioral, technological, and organizational components” [3]. The interplay between these various parts is often non-linear and unclear, making the implementation and evaluation inherently difficult [4,5]. The current project seeks to develop and test a scientifically grounded implementation strategy for a complex health intervention designed to serve the chronically homeless known as the Housing First model (HFM). As with most housing interventions for the homeless, Housing First requires interaction among multiple individuals (e.g., providers, case managers, landlords), organizations (e.g., government funders, non-profit service providers, property management), and systems (e.g., housing, medical, mental health, substance abuse) to be successful, thus making it a highly complex intervention to implement [6-9].

We propose to accomplish our goal by modifying a promising but limited HFM implementation strategy known as the Housing First Technical Assistance and Training Program (HFTAT) and testing its effectiveness among a small sample of Housing First programs. The protocol outlined in this paper describes both the development and research arms of this project, for which we are currently in the initial phases (i.e., the modification of the HFTAT). Specific questions guiding the research arm include the following: Is the adapted HFTAT a feasible implementation strategy for the HFM?; Does the adapted HFTAT lead to changes in implementation outcomes?; and How does the context of the intervention affect the implementation process? Before presenting our study protocol, we provide background related to the HFM and the original version of the HFTAT, describe the HFTAT’s limitations and how we plan to address them, and present the theoretical framework guiding our study design.

### Overview of the HFM and problems with implementation

The HFM is an evidence-based practice (EBP) demonstrated to lead to significant improvements for individuals who are chronically homeless, including higher housing retention [10,11] and improved physical and behavioral health [12-14]. The model was developed in the early 1990s to address the inadequacies related to serving chronically homeless clients existing in what have been referred to as “treatment first” programs [15]. These programs require consumers to demonstrate housing readiness through such things as sobriety, medication compliance, attainment of employment, and/or service engagement. In contrast, Housing First programs provide people immediate access to long-term housing with no precondition and very few service requirements [15].

Endorsements by national organizations have resulted in a rapid, nation-wide diffusion of the HFM since 2000 [16,17]. As this model has expanded, it has proven difficult to implement for a number of reasons, including a lack of replication guidelines during its initial diffusion [18] and contextual barriers (e.g., funding requirements, structure of available housing, pervasiveness of education in treatment first practices among staff) [19-21] and because the complexity of housing interventions requires significant coordination between multiple levels and systems to be successful [6-9]. Perhaps the most important of these reasons is the pervasiveness of abstinence-only attitudes among those who provide housing services, which can lead to poor model fidelity when resistance among administrators and/or staff occurs during the implementation process [19,21].

### The Housing First Technical Assistance and Training Program

The HFTAT was developed by the Midwest Harm Reduction Institute of Heartland’s Center for Systems Change (hereafter referred to as Heartland). It is a “blended implementation strategy”, employing face-to-face technical assistance and training, which includes a number of additional smaller strategies including the following: readiness and barrier assessment, identification and training of implementation leaders, implementation plan tailoring, building buy-in, and development of quality monitoring tools and systems [1]. The entire HFTAT delivery lasts 6 months to 2 years depending upon an organization’s needs. Technical assistance is provided to implementation leaders (typically administration, management, and/or key staff) through regularly scheduled meetings. These leaders are also provided with an implementation package that includes reading materials and tools for working with consumers. Technical assistance begins before training activities so the unique needs of the organization can be recognized and addressed. Subsequent technical assistance meetings are scheduled monthly to address implementation barriers and to develop policies and quality monitoring plans. Training is provided to administration and all staff who have direct contact with clients. Additional staff are welcome to participate in training depending on the organization’s goals/needs.

Findings from an early process evaluation conducted by external evaluators indicated the HFTAT led to a number of positive changes in two participating programs’ practices, policies, and staff attitudes [22]. Adherence and attitude data collected by Heartland staff from 14 programs as part of internal HFTAT evaluation activities also demonstrate the implementation strategy’s potential through its ability to change adherence and attitude scores between baseline and 1-year post-HFTAT delivery. Despite its initial promise, the HFTAT is limited as an implementation strategy because the face-to-face method of delivery requires a significant amount of coordination and resources [23]. We propose to address this limitation by modifying the HFTAT so it can be delivered across distance. This approach will utilize technical assistance activities facilitated by phone and online conferencing technology and the delivery of training through an e-learning platform.

### E-learning as organizational training

E-learning is a popular training method utilized by many large companies when implementing new policies and procedures. Well-designed e-learning can have significant advantages over face-to-face training due to the potential for higher levels of efficiency, flexibility, cost effectiveness, and ability to improve work behaviors [24-26]. An additional benefit of using e-learning as an implementation strategy is that it provides a psychologically safer way for individuals to interact with new concepts and tools that contrast with the existing personal and professional values of employees—as is often the case when staff with a treatment first work background are learning to work in the HFM. This dissonance can result in resistance to learning and change, particularly in face-to-face learning encounters [27]. In these situations, a benefit of well-designed e-learning is that learners have the opportunity to explore a new practice at their own pace in a private setting [28].

Anderson has proposed a model that is a useful guide for those seeking to effectively integrate e-learning within an implementation strategy [28]. This model requires balance between four attributes of effective learning: (1) strategies must be learner-centered, i.e., designed with respect for the learners’ prior experiences, culture, and work context and give them greater control over the educational experience [24,25,28,29]; (2) strategies must be knowledge-centered, i.e., learning that is relational, actively constructed, intentional, reflective, authentic, and contextualized [25,28-30]; (3) strategies must be assessment-centered, i.e., provide opportunities for learners to share their thinking at various stages of the learning process and receive meaningful feedback [28-30]; (4) and strategies must be community-centered, i.e., support the social construction of knowledge, the development of a learning community, and connect the content to the learner’s larger community and culture [29]. Continued participation in an active community of practice (i.e., others carrying out similar work) is key to supporting commitment to change after the formal training has been completed [25,28].

### Preliminary work related to this project

Dr. Watson conducted the external process evaluation of the HFTAT mentioned above [22]. He also developed the HFM Fidelity Index in collaboration with Heartland staff. When validating this instrument, Watson et al. [21] found wide variation in Housing First implementation, largely due to (1) the pervasiveness of treatment first and abstinence-only practices and (2) the high degree of complexity associated with the intervention. These findings underscore the need for a well-designed HFM implementation strategy. Our team also conducted an exploratory survey in August 2013 to gauge interest in participating in an online learning (and other topics) among housing providers. We received 195 responses from a convenience sample using established housing networks and mailing lists. Seventy-seven percent of respondents indicated interest in participating in an online community of practice to improve their knowledge of HFM practice.

### Potential impact to the field

Relatively little work has been carried out within the field of implementation to understand strategies aimed at putting EBPs into practice [1,2]*.* As such, this project will increase limited scientific knowledge regarding implementation strategies. Specifically related to the HFM, this project is an important first step in the development of an evidence-based implementation strategy that will increase the scalability and ultimate impact of the HFM. Given how ubiquitous the model has become and the demonstrated problems related to implementation, the development of such a strategy is in the best interest of policy makers, funders, providers, clients, and researchers.

### Theoretical framework

The theoretical framework guiding the overall project is a combination of two separate models: the first is a model proposed by Proctor, Landsverk, Aarons, Chambers, Glisson, and Mittman [2], and the second is proposed by Chaudoir, Dugan, and Barr [31]. We have chosen to integrate the two models because (1) Proctor et al. explicitly consider implementation strategies; (2) Chaudoir et al. specifically highlight the importance of the structural-levels (e.g., external systems and the community) important to consider with complex interventions such as Housing First; and (3) both models define implementation outcomes as distinct from both service and client outcomes, making them highly compatible.

**Figure 1:**
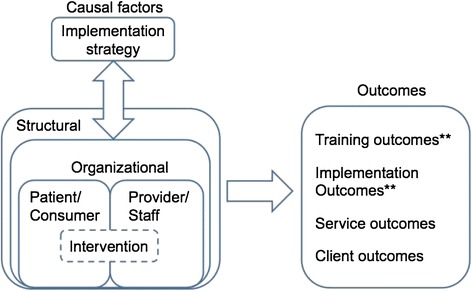
**Multi-level conceptual framework for predicting implementation outcomes of complex interventions.** Adapted from Chaudoir et al. [31] and Proctor et al. [2]. Double asterisks represent outcomes of interest current study.

The combined framework recognizes that implementation can occur separately or simultaneously at one or more levels (e.g., system, organizational, group, individual) and that appropriately targeted implementation strategies should lead to effective change. As demonstrated in Figure 1, the framework proposes the following: (1) the implementation strategy (e.g., the HFTAT) affects various constructs at multiple levels within which the intervention is located, (2) constructs at these levels also affect the implementation strategy through barriers and facilitators existing within (as represented by the bi-directional arrows), (3) an intervention often has to be adapted to fit the broader context in which it is situated (represented by the dashed lines), and (4) changes at these levels have effects on a variety of outcomes. As noted in Figure 1, this study will specifically measure the modified HFTAT’s ability to affect training and implementation outcomes.

## Methods/design

The first phase of the project, which began in July 2014, is focused on the adaptation of the HFTAT. The second will study the effectiveness of the HFTAT as an implementation strategy among four housing organizations. We estimate it will take 16 months to completely modify the HFTAT and an additional 20 months to test it. Indiana University’s Institutional Review Board has approved all research-related procedures described below.

### Adaptation of the HFTAT

The adapted HFTAT will encompass two main types of activities. (1) Technical assistance will be provided at the organizational level through initial consultation activities and through monthly check-ins with administrative staff. (2) Training activities will happen at the staff-level and will consist of didactic and interactive learning activities, assessment activities with formative and summative feedback, and engagement in an active online community of practice. The adapted HFTAT will be delivered over a 12-month period; the technical assistance portion will last the entire year, while training activities will last a maximum of 6 months.

#### Adaptation of technical assistance activities

The technical assistance component will utilize the following: (1) the HFM Fidelity Index (developed previously by members of the project team) to facilitate initial discussions with organizations, tailor an implementation plan, and track progress; (2) an expanded implementation package including the previously mentioned HFM Fidelity Index, templates for HFM policies and protocols, informational materials for consumers and stakeholders, and an implementation manual; and (3) video conferencing and telecommunications technologies to facilitate monthly technical assistance meetings with implementation leaders, thus making the HFTAT more versatile as a training option. Fidelity instruments and implementation packages have both been used successfully to facilitate implementation in previous work [32].

#### Adaptation of the training

The goal of training activities is to support implementation as meaningful learning and commitment to change. Meaningful learning is actively constructed and intentional [33]. The challenge is in taking quantities of presentational material and related face-to-face activities and developing interactive e-learning activities to provide context, challenge, activity, and feedback while following appropriate principles of multimedia learning to support higher levels of cognition [34]. The diversity in the HFTAT curriculum offers opportunities to provide a mix of self-paced and asynchronously facilitated learning activities to sustain learner interest and motivation.

The adapted training will be delivered in four modules: (1) overall introduction to the HFM, (2) running a HFM program (for administration and implementation leaders only), (3) housing case management, and (4) strategies (largely clinical) for working with consumers. Learners will be instructed to complete the training largely at their own pace, though there will be specified dates by which they are expected to complete individual modules. Building on the affordances of available technology and utilizing Anderson’s model of e-learning as a guide, we are developing a combination of didactic materials and interactive learning activities (knowledge- and assessment-centered) that recognize both the individual and structural opportunities and challenges to implementation (learner-centered) and integrate a supportive, online, nation-wide community of practitioners (community-centered) to support participants in making and keeping their commitment to change.

Learner engagement and the provision of an active learning experience will be facilitated primarily through the utilization of two strategies. (1) We are integrating case-based narratives that will allow learners to explore the immediate utility of HFM concepts, tools, and practices [35]. Narratives will not be presented as a whole, but will be cut into smaller segments and threaded throughout the training where they best serve to reinforce specific concepts. (2) We will also provide opportunities for learner engagement in an active community of practice by providing a virtual space that will be open to all HFTAT participants, as well as individuals not participating in the training but working in the field of housing (not just those working in HFM programs). Similar approaches have been demonstrated to have a positive impact on the implementation and sustainability of EBPs [36,37]. The community of practice will provide a virtual space for social and collaborative learning, making information presented in the HFTAT more meaningful by embedding it within the larger HFM conversation [28]. It will also serve as a resource for implementation leaders to gain technical assistance beyond the end of the HFTAT, thus increasing the potential sustainability of the implementation strategy.

We are also utilizing the following additional strategies to facilitate a meaningful and engaging learning experience: cognitively effective design that will break longer topics into smaller, learner-controlled segments including a mix of audio, images, text, and video [38]; branched learning scenarios that allow the learner to influence content based on their choice of options available; providing learners with opportunities to put skills and knowledge gained into practice through authentic, performance-focused challenges, activities, and assessments, which will receive individualized feedback from training staff; and providing opportunities for reflection on prior work activities within a treatment first model and the assumptions on which they were based to support conceptual change [30].

#### Alpha testing of training modules

We will conduct an alpha test of each training module and make necessary refinements before testing the full HFTAT as an organizational implementation strategy. We are currently recruiting front-line staff working in housing programs to participate in the alpha test using a snowball sampling approach. We are recruiting participants from programs that self-designate as Housing First and treatment first so our data represent experiences of both HFM experts and neophytes. We plan to recruit a total of 10 participants with a range of HFM work experiences (from no experience to substantial experience). We will recruit five participants from the City of Chicago and five from Central Indiana (the two areas where the full implementation strategy will be tested; see below). To best understand the effectiveness of the training under “real world” conditions, we will ask participants to complete the modules in a setting comfortable to them using their own equipment.

Participants will keep a detailed log as they independently work through each module and engage with the community of practice, an approach often used to understand user experience of new technologies [39]. We will instruct them to use a form provided with specific spaces to record: (1) questions they have on the content, presentation, and assessment; (2) any technical issues they may experience; and (3) general thoughts and affective responses to the material and activities. The open-ended question regarding general thoughts and responses will allow us to capture concerns and ideas unforeseen in our instrumentation which emerge from the user experience. We will also conduct one focus group with users in each city (two focus groups total), which will allow participants to respond to all comments and feedback received, thus eliciting a variety of views on the material [40]. Interview guides for focus groups will be structured similarly to the logs in terms of querying content, presentation, assessment, and experiences of technology. We will develop additional queries and probes from early analyses of log data (see “Analytic strategy” section). Participants will receive $100 for each module they complete and $30 for the focus group ($430 total per participant).

### Analytic strategy

As is typical in an inductive approach, data analysis will be ongoing and iterative. We will utilize MAXQDA, a qualitative analysis program, to assist us with the categorization and analysis of data [41]. The initial analysis of log data will allow us to incorporate experiences described by users as additional themes for discussion in focus groups. Thereafter, we will analyze log and focus group data as a single dataset (i.e., we will combine both individual and group-level data). We will employ directed content analysis, a technique whereby themes will be initially identified and coded in data as they pertain to questions we asked about the usability of the modules, while still remaining open to the addition of new themes should they emerge [42]. This is important because, while we are knowledgeable about the subject matter addressed in the modules, we cannot predict all the ways in which others will experience the material *a priori*. We will summarize the themes identified through the data analysis and modify the modules based on the results before pilot testing begins.

### Testing of the adapted HFTAT

We will utilize a convergent parallel, mixed methods design to test the adapted HFTAT among a sample of four housing programs [43]. This design involves the concurrent but separate collection of both qualitative and quantitative data. The use of mixed methods is common practice in implementation research due to the complexity of implementation, the multiple levels of an organization involved, and the importance of understanding how process affects implementation outcomes.

Organizations will be purposefully selected so they are unique enough from each other to ensure findings are related to the implementation strategy and not similarities related to structural- or organizational-level factors. We are currently in the process of selecting two organizations from Chicago and two from Central Indiana, two areas that are extremely different in their receptiveness to the HFM. Chicago is very receptive to the HFM, as the city has had a Plan to End Homelessness based on Housing First principles in effect since 2003. Indianapolis faces several barriers to HFM implementation, most importantly a reliance on Medicaid funds requiring service engagement that is not compatible with the principle of consumer choice emphasized by the HFM. We are also selecting the programs so they have different levels of familiarity with the HFM (i.e., HFM neophytes and programs seeking to improve their HFM practice). Due to the small size of some housing programs, we are only including those with 10 or more employees with direct client interaction as part of their job duties (e.g., case managers, program assistants, admissions staff, etc.). We are also requesting that members of the administrative team participate in the technical assistance portion of the HFTAT and associated data collection activities.

We will collect data reflecting staff characteristics such as demographics, job title, type of degree, primary discipline, length of time providing housing services, and length of time in current position. Agency characteristics we will collect include location, clients served, number of staff, length of time in existence, type of housing offered (single- or multiple-site), and primary source of funding.

The rest of our measurement selection is guided by the conceptual framework depicted in Figure 1. As such, measurement will focus on three main areas: causal factors, training, and implementation.

We will utilize the following three measures to assess causal factors hypothesized to affect implementation existing at multiple levels within which the intervention is embedded. (1) We are currently developing an instrument to measure the structural-level factors affecting implementation, as we have been unable to find any preexisting measures suitable for this task. The development of this instrument will be described in a separate paper. (2) Organizational-level and provider/staff-level factors will be measured using the context assessment portion of the organizational readiness to change assessment (ORCA). The ORCA is designed to assess organizational-level variables believed to affect implementation that has tested positively for both inter-rater and convergent/discriminant validity (C. Helfrich, personal communication, August 30, 2013) [44]. The context assessment portion of the instrument is comprised of 23 five-point Likert-type items. (3) We will measure patient/consumer-level factors using items we have developed for this purpose, which we will insert at the end of the ORCA. Four questions are preceded by a stem: “In the past year, how frequently have you observed clients in your organization: (a) express belief that current practice patterns can be improved; (b) encourage and support change in practice patterns to improve their care; (c) demonstrate willingness to participate in new programs or services; (d) cooperate with staff and management when there are changes in services, practices, or procedures that affect them?” Respondents will be asked to rate the questions using the same five-point scale as the ORCA questions.

We will also collect data for the purposes of assessing outcomes directly related to the training and technical assistance provided through the HFTAT. The learning management system that will host the training will track (1) frequency of visits to training and (2) time spent in e-learning at the staff-level to understand the use and access patterns of learners and the time engaged in learning activities and use patterns. (3) We will measure cost at the staff-level by multiplying the number of hours providers engaged in training by staff hourly pay and fringe rates. (4) A summative test delivered to staff at the end of the HFTAT will measure HFM knowledge (exact questions for this test will not be determined until the HFTAT is fully adapted). (5) We will assess satisfaction with training using 12 items from the Training Satisfaction Rating Scale [45]. We have selected 12 items from this instrument demonstrated to load the highest on three training dimensions: objectives and content, method and training context, and usefulness and overall rating. Each question is assessed using a five-point (1 = totally disagree through 5 = totally agree) Likert-type scale. The questions are general enough to be used to assess a wide array of trainings. (6) We will assess overall satisfaction with HFTAT using data collected through semi-structured phone interviews conducted with implementation leaders. These interviews will cover questions such as the following: How helpful did you find the initial implementation planning?; How helpful were the monthly technical assistance meetings?; How do you suggest the technical assistance portion of the HFTAT could be improved?; and How helpful was the training at preparing your employees to work in a HFM?

We will also assess 4 implementation outcomes. (1) We will measure fidelity using the HFM Fidelity Index. The index comprises 29 elements. Each element is scored regarding the degree to which it has been implemented along a scale that contains five descriptive anchors (1 = weakest level of implementation through 5 = strongest level of implementation) and has demonstrated construct and discriminant validity [21]. A series of interview questions are used to collect information necessary for identifying the correct anchor through a structured phone interview. (2) We will utilize the stages of implementation completion (SIC) instrument to measure implementation process and organizational change. [46] The SIC is an assessment tool comprising 31 items which measures and monitors completion of key activities related to implementation and the length of time to complete them. While still in development, there is evidence supporting the SIC’s reliability and ability to predict implementation success [47]. (We are currently working with the developers of the SIC to adapt it for use with the HFM.) Heartland staff will update the SIC through information gained in monthly technical assistance meetings. (3) The Evidence-Based Practice Attitude Scale measures acceptability of the intervention [48]. This scale has 15 general questions asking respondents to state the extent to which they agree with a set of questions along a four-point Likert-type scale in order to understand their attitudes towards the adoption of a new intervention. (4) Finally, we will conduct individual interviews to assess a number of other implementation outcomes including feasibility (i.e., usefulness of an EBP in a particular setting), appropriateness (i.e., perceived fit with the organization), adoption (i.e., intention to employ an EBP), and penetration (i.e., the degree to which staff have implemented HFM practices in their daily work). The interviews will cover questions such as the following: How is/has the move to the HFM affecting/affected your work?; How compatible is the HFM with your organization?; How interested are you in learning and applying what you will learn in the HFTAT training?; and How are you integrating what you learned in the HFTAT into your work?

Table 1 displays the time point(s) at which we plan to collect data related to each measure. Measures of causal factors will be collected at baseline. Data related to training outcomes will be collected from staff and implementation leaders after the training is completed, though HFM knowledge will also be measured at 12 months. Overall satisfaction with the HFTAT will be measured at 12 months. Regarding implementation outcomes, all measures with the exception of the HFM Fidelity Index and the SIC will be collected at baseline, immediately following training, and at 12 months. We will measure fidelity at baseline and 12 months. Due to the nature of the instrument, we will collect SIC data on a monthly basis.

**Table 1:** **Summary of data collection procedures for HFTAT testing**

**Measure**	**Method of data collection**	**Data source**	**Construct type**	**Data collection schedule**
• Structural-level	Electronic	Staff	Causal factor	Baseline
• Org- and staff-level				
• Consumer-level				
• Visit frequency	Electronic	Staff	Training outcome	After training
• Completion time				
• Cost				
• Training satisfaction				
HFM knowledge	Electronic	Staff	Training outcome	• After training
				• 12 months
Overall satisfaction with HFTAT	Phone interview	Implement leaders	Training outcome	12 months
Fidelity	Phone interview	Implement leaders	Implement outcome	• Baseline
				• 12 months
Implementation process (SIC)	Collected ongoing through technical assistance activities	Implement leaders	Implement outcome	N/A
Acceptability	Electronic	Staff	Implement outcome	• Baseline
				• After training
				• 12 months
• Feasibly	Individual interview	Staff	Implement outcome	• Baseline
• Appropriateness				• After training
• Adoption				• 12 months
• Penetration				

Because staff will most likely be required by their administration to go through the training as part of their organization’s commitment to HFM implementation, it is important to separate training and research activities. Participation in data collection related to frequency of visits to training, e-learning activity completion time, and HFM knowledge will be required as part of participation in training activities. Participation in all other data collection activities will be voluntary. For each instrument completed, we will enter staff participating in the collection of electronic data into a drawing for their organization to win one of two $50 gift certificates to a retailer or restaurant of their choosing at each data collection point. We will enter names into the drawing for each instrument completed so participation on multiple instruments will increase the chances of obtaining the gift certificate. We will provide staff participants in focus groups with a $10 Starbucks gift card for their time (focus groups will occur during work hours, so participants will also be compensated by their agency). We will not invite administrators, managers, and implementation leaders to participate in focus groups to ensure staff feel comfortable sharing information.

### Analytic strategy, quantitative

The primary quantitative outcome of interest at the organizational-level is fidelity. We will compare fidelity scores at baseline and 12 months to gauge improvement. Mean and standard deviation of the improvement will be calculated. The implementation process and organizational change, measured by the SIC at the organizational level, are collected through the monthly technical assistance activities. For each organization, we will examine average improvement in SIC scores using a linear regression model and summarize the improvement across organizations using mean and standard deviation.

Acceptability of the intervention is measured at the staff level. The change in acceptability after training and at 12 months compared to the baseline will be calculated for each staff member and summarized using mean and standard deviation for each organization and across organizations.

At each time point, proportions will be reported for categorical variables and mean and standard deviations will be reported for continuous variables. Improvement on these outcomes is then reported by comparing the after-training and 3-month follow-up measures to the baseline measures.

### Analytic strategy, qualitative

We will follow the similar qualitative data analysis procedure to that described for the alpha testing of the modules (i.e., employing MAXQDA to assist with a directed content analysis [42]). We will also investigate differences and similarities in themes within and across organizations [49,50]. Because analysis will be ongoing during this phase, it will be important to test hypotheses and theories developed in earlier analyses against ongoing evidence [51]. As they develop, we will share themes with Heartland staff to strengthen conclusions drawn from the data. We will also check findings against emerging data as analysis continues. The overall analytic goal is to understand pre-implementation and post-implementation differences in order to develop a theory of how the HFTAT affects implementation processes.

### Mixing of qualitative and quantitative data

We will merge quantitative and qualitative data strands for a combined analysis after separate analyses have been carried out for each [43]. Given the small sample size, qualitative data will assist us in understanding potential effects of the intervention where quantitative data do not. Validity will be strengthened should quantitative and qualitative results converge [40,43].

## Discussion

Based on previous performance of the HFTAT in its face-to-face format, we expect the adapted version will be evaluated positively by administrators and staff. We also anticipate that a combination of didactic and interactive learning materials, which recognize both the individual and structural opportunities and challenges to implementation and a supportive, online, nation-wide community of practitioners, will support participants in making a commitment to change during the HFM implementation process [25,28-30].

We plan to use findings from this project to inform a larger study to investigate the effectiveness of the adapted HFTAT as a strategy leading to sustainable implementation in comparison to alternative approaches among a larger, national sample of organizations. We will refine data collection instruments and protocols for this larger study based on our results. Because implementation of a complex EBP like the HFM affects all levels of an organization, we are also interested in examining the effect of implementation on client and service outcomes in this future study.

It is our intent that this project and the subsequent one will result in an evidence-based implementation strategy that will increase the scalability and ultimate impact of the HFM. The development of such a strategy is in the best interest of policy makers, funders, providers, clients, and researchers, given how ubiquitous the model has become and the demonstrated problems related to its implementation. The proposed projects will also increase limited scientific knowledge regarding implementation strategies [2,52], as well as provide an opportunity to test the generalizability of tools originally created to measure implementation of specific EBPs (e.g., the ORCA and the SIC).

## Abbreviations

HFM: Housing First model
HFTAT: Housing First Technical Assistance and Training Program
EBP: evidence-based practice
ORCA: organizational readiness to change assessment
SIC: stages of implementation completion
